# Renal Manifestations and their Association with Mortality and Length of Stay in COVID-19 Patients at a Safety-net Hospital

**DOI:** 10.2478/jccm-2022-0010

**Published:** 2022-05-12

**Authors:** Sandra Gomez-Paz, Eric Lam, Luis Gonzalez-Mosquera, Diana Cardenas-Maldonado, Joshua Fogel, Ellen Gabrielle Kagan, Sofia Rubinstein

**Affiliations:** 1 Division of Nephrology and Hypertension, Department of Internal Medicine, Nassau University Medical Center, East Meadow, New York, USA; 2 Department of Internal Medicine, Nassau University Medical Center, East Meadow, New York, USA; 3 Department of Business Management, Brooklyn College, Brooklyn, New York, USA

**Keywords:** acute kidney injury, COVID-19, length of stay, mortality, SARS-CoV-2

## Abstract

**Background:**

Renal involvement in COVID-19 leads to severe disease and higher mortality. We study renal parameters in COVID-19 patients and their association with mortality and length of stay in hospital.

**Methods:**

A retrospective study (n=340) of confirmed COVID-19 patients with renal involvement determined by the presence of acute kidney injury. Multivariate analyses of logistic regression for mortality and linear regression for length of stay (LOS) adjusted for relevant demographic, comorbidity, disease severity, and treatment covariates.

**Results:**

Mortality was 54.4% and mean LOS was 12.9 days. For mortality, creatinine peak (OR:35.27, 95% CI:2.81, 442.06, p<0.01) and persistent renal involvement at discharge (OR:4.47, 95% CI:1.99,10.06, p<0.001) were each significantly associated with increased odds for mortality. Increased blood urea nitrogen peak (OR:0.98, 95%CI:0.97,0.996, p<0.05) was significantly associated with decreased odds for mortality. For LOS, increased blood urea nitrogen peak (B:0.001, SE:<0.001, p<0.01), renal replacement therapy (B:0.19, SE:0.06, p<0.01), and increased days to acute kidney injury (B:0.19, SE:0.05, p<0.001) were each significantly associated with increased length of stay.

**Conclusion:**

Our study emphasizes the importance in identifying renal involvement parameters in COVID-19 patients. These parameters are associated with LOS and mortality, and may assist clinicians to prognosticate COVID-19 patients with renal involvement.

## Introduction

Coronavirus Disease 2019 (COVID-19) caused by the novel severe acute respiratory syndrome coronavirus 2 (SARS-CoV-2) affects mainly the respiratory system, but extrapulmonary manifestations occur including renal involvement [1, 2]. Renal involvement has an incidence rate ranging from 25-67% in COVID-19 patients [3, 4, 5]. Acute kidney injury (AKI) is a frequent complication in patients with COVID-19 and is associated with intensive care unit (ICU) admission rates of over 50% [4] and increased mortality rate [1, 5].

Risk factors for renal involvement in patients with COVID-19 include older age, African American race/ ethnicity, hypertension, diabetes mellitus, cardiovascular disease, invasive mechanical ventilation, and vasopressor use [6]. In patients with COVID-19 and AKI, invasive mechanical ventilation is associated with higher mortality [6, 7]. In COVID-19, renal involvement varies from AKI stage 1 to requiring renal replacement therapy [1, 4]. However, other disease severity indicators such as other organ system involvement and persistent renal involvement at discharge and their association with mortality and length of stay (LOS) in patients with COVID-19 and renal injury have not been described.

The aim of our study is to investigate previously studied renal parameters of creatinine peak level, BUN peak level, need for renal replacement therapy, and also previously not studied renal parameters of days-to-renal involvement, total number of other organs

involved, and persistent renal involvement at the time of discharge or death in COVID-19 patients with renal involvement and their association with mortality and LOS. We hypothesize that these previously not studied variables are associated with increased mortality and LOS in COVID-19 patients with renal involvement.

## Methods

### Study Design and Participants

We conducted a retrospective study of 340 patients with confirmed COVID-19 infection and renal involvement that were admitted to our safety-net institution in a suburban New York City hospital from March 1,2020 to May 15, 2020. A positive result from a nasopharyngeal sample of real-time reverse transcription polymerase chain reaction (RT-PCR) result for SARS-CoV-2 confirmed positive COVID-19 infection. Renal involvement was determined by the presence of acute kidney injury (AKI) as per Kidney Disease Improving Global Outcomes (KDIGO) Guidelines [8]. All participants completed their hospital course as discharged alive or deceased. The hospital Institutional Review Board granted ethical approval to conduct this study. A waiver for informed consent was obtained due to the retrospective nature of the study.

### Variables

Demographic characteristics were age (years), sex (male, female), race/ethnicity [Caucasian, African American, Hispanic and/or Latino, and Other], and insurance status (private, uninsured/emergency Medicaid, regular Medicaid, and Medicare). Comorbidities were obesity (BMI30.0 kg/m^2^) and the Charlson Comorbidity Index (CCI) which includes a range of comorbid conditions of age, renal disease, diabetes mellitus, history of myocardial infarction, heart failure, peripheral vascular disease, chronic obstructive pulmonary disease, dementia, cerebrovascular accident, solid tumor, leukemia, lymphoma, AIDS status, chronic liver disease, connective tissue disease, and peptic ulcer disease (37 points total) [9]. The CCI predicts 10-year survival [9].

Indicators for disease severity were the admission qSOFA (Quick Sequential Organ Failure Assessment) score (3 points total) [10], ICU admission, intubation at the time of admission, and oxygen requirement during hospitalization [11]. ICU admission was defined by the need for supplemental oxygen with fraction of inspired oxygen (FiO2) >55% (with or without invasive mechanical ventilation) or use of vasopressor medications. Oxygen requirement during hospitalization was divided as none or low FiO2 (<55%) requirement, high FiO2 requirement (>55%), and the need for invasive mechanical ventilation (intubation).

Renal involvement was defined by the presence of AKI as per KDIGO guidelines, in which AKI is defined as an increase in serum creatinine level (SCr) by 0.3 mg/dl within 48 hours of admission or 1.5 times increase in SCr from known baseline or presumed to have increased within the prior 7 days [8]. AKI was classified per Acute Kidney Injury Network (AKIN) criteria as stage 1 (increase in SCr > 0.3mg/dL or 1.5-times from baseline), stage 2 (two times increase in SCr 2 from baseline), and stage 3 (increase in SCr > 4mg/dL with acute rise of >0.5mg/dL or three times increase in SCr from baseline or new renal replacement therapy, irrespective of SCr) [8]. Days-to-renal involvement was defined as the total number of days from admission until the first day of renal involvement. Persistent renal involvement at discharge was defined by persistent AKI or failure to return to baseline SCr on the last day of hospitalization. Other renal involvement variables included creatinine peak level (mg/dl), blood urea nitrogen (BUN) peak level (mg/dl), serum sodium level on admission (mEq/L), serum calcium level during hospitalization (mg/dL), urine specific gravity, and new renal replacement therapy (NRRT) during hospitalization (no/yes).

Treatment/management variables measured as no/ yes were the use of vasopressor medication, any antibiotic, antiviral (remdesivir), antimalarial (hydroxychloroquine or chloroquine), non-steroidal anti-inflammatory drugs (NSAIDs), angiotensin-converting-enzyme inhibitors (ACE-i) or angiotensin-II-receptor blockers (ARBs), any steroid medication, convalescent plasma from COVID-19 survivor donors, interleukin inhibitors (anti-IL6 monoclonal antibodies-tocilizumab), and therapeutic dosage of anticoagulant medications.

The number of organs involved was defined as the sum of eight organs that were impaired or involved during admission. Involvement of each organ was determined by collecting the following information: mean arterial pressure (MAP) on admission (mmHg) for the cardiovascular system; Glasgow Coma Score (GCS) nadir during hospitalization for the neurological system; nadir oxygen saturation <95% or any new requirement of supplemental oxygenation from baseline for respiratory involvement; peak total bilirubin level (mg/dL) for the gastrointestinal/liver system; peak creatinine level (mg/dL) for the renal system; peak blood glucose level (mg/dL) for the endocrine system; nadir white blood cell (k/mm^3^) and peak platelet level (k/mm^3^) for the hematologic system; peak creatine kinase level (U/L) for the musculoskeletal system. The primary outcome was mortality (no/yes) and the secondary outcome was length of stay (LOS) in the hospital (days).

### Statistical Analysis

Descriptive statistics consisted of mean and standard deviation for the continuous variables and frequency and percentage for the categorical variables. Two models were used to analyze the outcome variables of mortality and LOS. Univariate analyses were used for Model 1 that considered demographic, comorbidities, disease severity, and treatment management variables. A multivariate analysis was used for Model 2 that included all the significant variables from the univariate analyses in Model 1 and added respiratory variables. Mortality was analyzed with logistic regression. LOS was analyzed with linear regression. Logarithmic transformations were performed for the skewed variables. All p-values were two-sided. Data analyses were conducted with IBM SPSS Statistics version 26 (IBM Corporation, Armonk, NY, 2019).

## Results

Table 1 shows the sample characteristics. Mean age was above 65 years, more than one-third were female, and 62.9% were those from either African American or Hispanic race/ethnicity. The mean CCI score was 3.6 (out of a possible 37 points) and the mean qSOFA score was 2.0 (out of a possible 3 points). Over 70% were admitted to ICU, more than 45% required invasive mechanical ventilation during hospitalization, and 32.1% required vasopressors. The most common treatments were antibiotics (95.6%) and antimalarial (86.5%). Mean SCr peak was 3.6, mean BUN was 72.7, mean calcium was 8.3, and mean sodium was 139.0. Mean days-to-renal involvement was approximately 3 days. More than 13% of patients required new renal replacement therapy during hospitalization and almost 60% had persistent renal involvement at the time of discharge. More than 34% of patients were at AKIN stage 3. Overall mortality was 54.4% and mean LOS was 12.9 days.

**Table 1:** Sample Characteristics of 340 COVID-19 Patients

Variables	M (SD) or Frequency (Percent)
Demographics	
Age (years) [mean]	65.7 (15.36)
Sex (female)	123 (36.2)

Race/ethnicity	
Caucasian	94 (27.6)
African American	97 (28.5)
Hispanic	117 (34.4)
Other	32 (9.4)

Insurance	
Private	67 (19.7)
Uninsured/Emergency Medicaid	56 (16.5)
Regular Medicaid	128 (37.6)
Medicare	89 (26.2)

Comorbidities	
Obese (yes)	128 (37.6)
CCI [mean]	3.6 (2.48)

Disease severity	
qSOFA [mean]	2.0 (0.66)
ICU (yes)	239 (70.3)
Intubation admission (yes)	35 (10.3)

Oxygen requirement hospitalization	
None/Low FiO2 (< 55%)	98 (28.8)
High FiO2 (> 55%)	88 (25.9)
Ventilation	154 (45.3)

Treatment management	
Vasopressor (yes)	109 (32.1)
Antibiotic (yes)	325 (95.6)
NSAID (yes)	85 (25.0)
ACEi/ARBS (yes)	44 (12.9)
Antiviral (yes)	11 (3.2)
Antimalarial (yes)	294 (86.5)
Steroid (yes)	134 (39.4)
Convalescent plasma (yes)	35 (10.3)

Interleukin inhibitor (yes)	47 (13.8)
Anticoagulant (yes)	91 (26.8)

Organ involvement	
Number organs involved [mean]	5.3 (1.54)

Renal	
Creatinine peak [mean]	3.6 (3.25)
Blood urea nitrogen peak [mean]	72.7 (53.13)
Calcium during hospitalization [mean]	8.3 (0.81)
Sodium on admission [mean]	139.0 (7.17)
Days to renal involvement [mean]	2.8 (3.98)

AKIN stage	
Stage 1	158 (46.5)
Stage 2	65 (19.1)
Stage 3	117 (34.4)
New renal replacement therapy (yes)	45 (13.2)
Renal persistent involvement at discharge	200 (58.8)
(yes)	
Specific gravity [mean]	1.02 (0.01)

Outcomes	
Mortality (yes)	185 (54.4)
Length of stay (days) [mean]	12.9 (13.18)

Note: M=mean, SD=standard deviation, CCI=Charlson Comorbidity Index, qSOFA=quick Sepsis Related Organ Failure Assessment, ICU=intensive care unit, FiO2=fraction of inspired oxygen, NSAID=nonsteroidal anti-inflammatory drug, ACEi=Angiotensin-converting-enzyme inhibitors, ARB=angiotensin II receptor blockers, AKIN=acute kidney injury network. Specific gravity only had data available from 210 patients.

Table 2 shows logistic regression analyses for mortality. In the multivariate analysis shown in Model 2, increased age, oxygen requirement during hospitalization of high FiO2 and ventilation, increased number organs involved, creatinine peak, and renal persistent involvement at discharge were each significantly associated with increased odds for mortality. African American race/ethnicity and increased blood urea nitrogen peak were each significantly associated with decreased odds for mortality.

**Table 2:** Logistic Regression Analyses for Mortality

**Variables**	**Model 1 - Univariate OR (95% CI)**	**Model 2 - Multivariate OR (95% CI)**
*Demographics*		
Age (years)	1.03 (1.01, 1.04)***	1.09 (1.05, 1.14)***
Sex (female)	0.86 (0.55, 1.34)	---

Race/ethnicity		
Caucasian	1.00	1.00
African American	0.52 (0.29, 0.92)*	0.34 (0.13, 0.91)*
Hispanic	0.70 (0.40, 1.22)	0.84 (0.29, 2.44)
Other	1.19 (0.51, 2.74)	0.62 (0.15, 2.53)

Insurance		
Private	1.00	---
Uninsured/Emergency Medicaid	1.55 (0.76, 3.16)	
Regular Medicaid	1.28 (0.71, 2.31)	
Medicare	1.88 (0.99, 3.57)	

*Comorbidities*		
Obese (yes)	0.92 (0.59, 1.43)	---
CCI	1.18 (1.08, 1.30)***	0.98 (0.78, 1.23)

*Disease severity*		
qSOFA	1.29 (0.93, 1.79)	---
ICU (yes)	19.44 (9.99, 37.84)***	0.91 (0.12, 7.08)
Intubation admission (yes)	4.62 (1.86, 11.44)**	0.45 (0.12, 1.67)

Oxygen requirement hospitalization		
None/Low FiO2 (< 55%)	1.00	1.00
High FiO2 (> 55%)	14.24 (5.93, 34.16)***	17.59 (1.98, 156.41)*
Ventilation	78.00 (31.98, 190.22)***	201.93 (15.60, 2,614.08)***

*Treatment management*		
Vasopressor (yes)	9.64 (5.26, 17.66)***	0.96 (0.30, 3.02)
Antibiotic (yes)	2.48 (0.83, 7.43)	---
NSAID (yes)	1.05 (0.64, 1.72)	---
ACEi/ARBS (yes)	0.54 (0.28, 1.02)	---
Antiviral (yes)	8.80 (1.11, 69.53)*	2.56 (0.19, 34.07)
Antimalarial (yes)	0.66 (0.35, 1.26)	---
Steroid (yes)	2.39 (1.52, 3.76)***	1.20 (0.48, 3.03)
Convalescent plasma (yes)	1.13 (0.56, 2.29)	---
Interleukin inhibitor (yes)	1.95 (1.01, 3.76)*	1.08 (0.34, 3.47)
Anticoagulant (yes)	1.03 (0.64, 1.67)	---

*Organ involvement*		
Number organs involved	2.71 (2.18, 3.38)***	1.83 (1.24, 2.71)**

*Renal*		
Creatinine peak	---	35.27 (2.81, 442.06)**
Blood urea nitrogen peak	---	0.98 (0.97, 0.996)*
Calcium during hospitalization	---	1.49 (0.93, 2.39)
Sodium on admission	---	1.03 (0.97, 1.09)
Days to renal involvement	---	1.21 (0.37, 3.93)
AKIN stage	---	
Stage 1		1.00
Stage 2		1.09 (0.34, 3.50)
Stage 3		0.84 (0.13, 5.59)
New renal replacement therapy (yes)	---	0.27 (0.07, 1.10)
Renal persistent involvement at discharge	(yes) ---	4.47 (1.99, 10.06)***

Note: OR=odds ratio, CI=confidence interval, CCI=Charlson Comorbidity Index, qSOFA=quick Sepsis Related Organ Failure Assessment, ICU=intensive care unit, FiO2=fraction of inspired oxygen, NSAID=nonsteroidal anti-inflammatory drug, ACEi=Angiotensin-converting-enzyme inhibitors, ARB=angiotensin II receptor blockers, AKIN=acute kidney injury network. *p<0.05, **p<0.01, ***p<0.001, Model 2 Nagelkerke R Square=0.75.

Table 3 shows linear regression analyses for LOS. In the multivariate analysis shown in Model 2, steroid, convalescent plasma, anticoagulant, increased number organs involved, increased blood urea nitrogen peak, NRRT, and increased days to renal involvement were each significantly associated with increased LOS.

**Table 3:** Linear Regression Analyses for Length of Stay

Variables	Model 1 - Univariate B (SE)	Model 2 - Multivariate B (SE)
*Demographics*		
Age (years)	-0.01 (0.001)***	-0.002 (0.02)
Sex (female)	-0.10 (0.04)*	-0.01 (0.04)

Race/ethnicity		---
Caucasian	Reference	
African American	<0.001 (0.05)	
Hispanic	0.09 (0.05)	
Other	-0.06 (0.07)	

Insurance		---
Private	Reference	
Uninsured/Emergency Medicaid	0.04 (0.07)	
Regular Medicaid	-0.09 (0.06)	
Medicare	-0.07 (0.06)	

*Comorbidities*		
Obese (yes)	-0.01 (0.04)	---
CCI	-0.03 (0.01)***	-0.004 (0.01)

*Disease severity*		
qSOFA	-0.05 (0.03)	---
ICU (yes)	0.19 (0.04)***	0.07 (0.08)
Intubation admission (yes)	-0.08 (0.07)	---

Oxygen requirement hospitalization		
None/Low FiO2 (< 55%)	Reference	Reference
High FiO2 (> 55%)	0.07 (0.05)	-0.09 (0.08)
Ventilation	0.21 (0.05)***	-0.14 (0.09)

*Treatment management*		
Vasopressor (yes)	0.15 (0.04)***	-0.05 (0.05)
Antibiotic (yes)	0.24 (0.10)*	0.12 (0.08)
NSAID (yes)	0.07 (0.05)	---
ACEi/ARBS (yes)	0.05 (0.06)	---
Antiviral (yes)	0.17 (0.11)	---
Antimalarial (yes)	0.07 (0.06)	---
Steroid (yes)	0.28 (0.04)***	0.10 (0.04)*
Convalescent plasma (yes)	0.49 (0.06)***	0.19 (0.06)**
Interleukin inhibitor (yes)	0.35 (0.05)***	0.02 (0.06)
Anticoagulant (yes)	0.26 (0.04)***	0.10 (0.04)**

*Organ involvement*		
Number organs involved	0.07 (0.01)***	0.04 (0.02)*

*Renal*		
Creatinine peak	---	-0.11 (0.11)
Blood urea nitrogen peak	---	0.001 (<0.001)**
Calcium during hospitalization	---	0.02 (0.02)
Sodium on admission	---	-0.01 (0.002)*
Days to renal involvement	---	0.19 (0.05)***
AKIN stage	---	
Stage 1		Reference
Stage 2		0.04 (0.05)
Stage 3		0.05 (0.08)
New renal replacement therapy (yes)	---	0.19 (0.06)**
Renal persistent involvement at discharge (yes)	---	-0.12 (0.04)**
Constant	---	1.28 (0.36)***

Note: B=unstandardized beta, SE=standard error, CCI=Charlson Comorbidity Index, qSOFA=quick Sepsis Related Organ Failure Assessment, ICU=intensive care unit, FiO2=fraction of inspired oxygen, NSAID=nonsteroidal anti-inflammatory drug, ACEi=Angiotensin-converting-enzyme inhibitors, ARB=angiotensin II receptor blockers, AKIN=acute kidney injury network. *p<0.05, **p<0.01, ***p<0.001, Model 2 adjusted R Square=0.37.

Increased serum sodium level on admission and increased renal persistent involvement at discharge were each significantly associated with decreased LOS.

## Discussion

We found partial support for our hypothesis for persistent renal involvement at discharge which was significantly associated with increased mortality but decreased LOS. We found full support for our hypothesis for increased number of organs involved which was significantly associated with increased mortality and increased LOS. We found partial support for our hypothesis for increased days to renal involvement which was significantly associated with increased LOS but not significantly associated with mortality. We found that the renal variable of increased BUN peak was associated with decreased odds for mortality and increased LOS. Creatinine peak level was significantly associated with increased mortality but now with LOS. Additionally, NRRT was significantly associated with increased LOS but not associated with mortality. Increased age was significantly associated with increased mortality. African American race/ethnicity was associated with decreased odds for mortality. CCI was not associated with mortality or LOS. The disease severity variables of oxygen requirement during hospitalization of high FiO2 and mechanical ventilation were each significantly associated with mortality but not LOS. Treatment with steroid therapy, convalescent plasma, and anticoagulant medications were associated with increased LOS but were not associated with mortality.

We found that renal involvement adjusted for all other organ involvement was associated with increased mortality and increased LOS. Multi-organ dysfunction is associated with increased mortality in critically-ill COVID-19 patients [12]. Renal injury is associated with higher mortality and severe disease in COVID-19 [3, 13]. Our study is the only study with COVID-19 patients with renal involvement adjusting for all other organ systems involvement and finding the same pattern of high mortality. Consistent with other studies [3, 13], our findings suggest that renal involvement is an independent and important prognostic factor in mortality in COVID-19 patients. We found that renal involvement adjusted for all other organ involvement was associated with increased LOS. This study is consistent with another study that reported that AKI in COVID-19 patients is associated with increased LOS [14]. The increased LOS can likely be explained by the added disease complexity where longer treatment time is needed to manage severe disease.

We found that increased SCr peak level was associated with increased mortality, whereas increased BUN peak level was associated with decreased mortality and increased LOS. Higher SCr peak levels in AKI are associated with high mortality in COVID-19 patients [6, 15]. Our results are consistent with these findings. BUN level is not specific to renal function, as it can be affected by many other conditions [16]. While some report that increased BUN level is associated with increased mortality [7, 15], others did not find that association [17]. We suggest that peak BUN is not an accurate predictor of mortality in COVID-19 patients with renal involvement. Peak BUN level and its association with LOS has not been studied in COVID-19. However, studies from other diseases reported that high BUN levels were associated with prolonged LOS [18]. Our study finding is consistent with this pattern. We suggest that patients with high BUN levels have additional pathological conditions requiring care and prolonging hospital stay.

We found that persistent renal involvement at discharge or death was associated with increased mortality and decreased LOS. There is one study that investigated renal involvement at discharge and found that 35% of patients discharged alive had persistent AKI [3]. Approximately 60% of our patients had persistent renal involvement at discharge or at death. Data regarding COVID-19 patients with renal involvement and LOS is mixed. In unadjusted models, some show increased LOS in patients with AKI and COVID-19 [19], while others do not show any association [20]. We found a decreased association after adjusting for relevant covariates. A study reported that hospitalized COVID-19 patients with higher mortality had decreased LOS [21]. We suggest that our high mortality level for COVID-19 patients with renal involvement is the reason for the decreased LOS.

We found that NRRT was associated with increased LOS. Others report that AKI in COVID-19 patients is associated with increased LOS [19], including those who received continuous replacement therapy [22]. Patients receiving NRRT have more severe disease requiring longer duration until stabilization and discharge. We did not find an association of NRRT with mortality in COVID-19 patients with renal involvement. Although some report that NRRT in all hospitalized COVID-19 patients is associated with increased mortality [3, 23], no study focused on COVID-19 patients with renal involvement. As COVID-19 patients with renal involvement is associated with increased mortality [3], the subgroup of NRRT renal patients is not likely to add any additional association for mortality.

We found that greater number of days to renal involvement was associated with increased LOS. The median time of incidence of AKI in COVID-19 patients is 1-3 days upon hospitalization [3, 24, 25]. A later onset time of AKI may correlate with severe disease, which is associated with increased LOS [26]. Another possible explanation is that later onset of renal involvement requires more time for treatment and monitoring thus prolonging the LOS.

We found that increased sodium levels on admission were associated with decreased LOS but there was no association with mortality. Most studies on hospitalized COVID-19 patients show that hyponatremia on admission is associated with worse prognosis, including higher ICU admission and mortality [27, 28], while this association is not consistent in hypernatremia [27, 28]. No studies investigated the admission sodium level among COVID-19 patients with renal involvement. Our finding suggests that admission sodium is not an independent risk factor for mortality in COVID-19 patients with renal involvement. We found that increased sodium level on admission was associated with decreased LOS. This finding is consistent with other studies, as COVID-19 patients with hyponatremia are associated with a higher rate of ICU admission [27, 28]. Although we did not find an association with AKIN stages and mortality, the proportion of patients in each category and the lack of association with mortality is consistent with other studies [3, 6]. We did not find an association of admission serum calcium with mortality or LOS, which is consistent with previous studies [29]. Our findings suggest that admission serum electrolytes of sodium and calcium may not be useful markers for COVID-19 disease severity in patients with renal involvement.

We found that increased age was associated with increased mortality in COVID-19 patients with renal involvement, which is consistent with others [20, 30]. We found that African American race/ethnicity had decreased odds for mortality. Although the incidence of renal failure in COVID-19 patients is higher in African Americans [31], data regarding the association between race/ethnicity and mortality in COVID-19 patients with renal involvement is conflicting. Some reported increased mortality in African Americans [19], while others did not find any association [4, 6]. African Americans with end-stage renal disease on dialysis have lower mortality [32]. The survival advantage of African Americans is associated with the presence of APOL-1 genetic variants even though the APOL-1 genetic variant is linked to greater incidence of kidney disease in this group [32]. This suggests that the presence of APOL-1 may have a protective effect in African American COVID-19 patients with renal involvement and results in decreased mortality. We did not find an association between insurance status and mortality or LOS in COVID-19 patients with renal involvement. To our knowledge, there are no studies describing insurance status and its association with mortality or LOS among COVID-19 patients with renal involvement.

CCI was not associated with increased mortality in the multivariate analysis. Although CCI >3 is associated with higher mortality in hospitalized COVID-19 patients [33], there is only one study regarding its association in COVID-19 patients with renal involvement which reports no association [34]. Our findings are consistent with this study [35]. CCI was designed to predict 10-year mortality due to chronic diseases, and was not tailored to predict mortality in COVID-19. We did not find an association between obesity and mortality. While increased mortality is reported in hospitalized COVID-19 patients with severe obesity (BMI>40), increased mortality is not reported in patients with obesity (BMI>30) [35]. Our finding for COVID-19 patients with renal involvement is consistent with previous research.

We found that oxygen requirement of high FiO2 and the need for invasive mechanical ventilation were associated with increased mortality. The need for oxygen support with high levels of FiO2 and invasive mechanical ventilation leads to high mortality in hospitalized COVID-19 patients [11, 36]. We add that this occurs in COVID-19 patients with AKI. We did not find any association of vasopressor use with mortality or LOS in our multivariate analyses. Although use of vasopressor medications increases the risk for developing AKI in COVID-19 patients [6], no mortality association was found in hospitalized COVID-19 patients [37]. Our findings for COVID-19 patients with AKI have a similar pattern.

Antiviral medication, steroid therapy, and treatment with convalescent plasma were not associated with increased mortality in the multivariate analysis. There are mixed data regarding the mortality benefit of these therapies in COVID-19 patients with renal involvement [38, 39]. These mixed findings can be explained by the study being performed in the early phase of the pandemic when no clear treatment guidelines were available. We found that treatment with steroids, anticoagulant medications, and convalescent plasma were associated with increased LOS. This is likely related to patients needing to complete the course of their treatment that prolonged their hospital stay.

There are several study limitations. First, as this was a retrospective study, we were not able to include urinary parameters since this was insufficiently collected. Second, our study was performed in a single center, safety-net hospital. However, the many minorities included provides a good perspective on the impact of renal involvement in COVID-19 in these racial/ethnic groups. Third, our study was performed during the early stages of the pandemic. Lastly, we did not include all variables that were associated to mortality. However, our study is adjusted for the most common factors - age, comorbidities, and oxygen requirement - contributing to increased mortality and LOS.

## Conclusion

Our study emphasizes the importance in identifying renal involvement parameters in COVID-19 patients. These parameters are associated with LOS and mortality, and may assist clinicians to prognosticate COVID-19 patients with renal involvement.

## References

[1] Pei G, Zhang Z, Peng J, Liu L, Zhang C, Yu C (2020). Renal involvement and early prognosis in patients with COVID-19 pneumonia. J Am Soc Nephrol.

[2] Lai CC, Ko WC, Lee PI, Jean SS, Hsueh PR (2020). Extra-respiratory manifestations of COVID-19. Int J Antimicrob Agents.

[3] Chan L, Chaudhary K, Saha A, Chauhan K, Vaid A, Zhao S (2021). AKI in hospitalized patients with COVID-19. J Am Soc Nephrol.

[4] Nadim MK, Forni LG, Mehta RL, Connor MJ Jr, Liu KD, Ostermann M (2020). COVID-19-associated acute kidney injury: consensus report of the 25th Acute Disease Quality Initiative (ADQI) Workgroup. Nat Rev Nephrol.

[5] Gupta S, Hayek SS, Wang W, Chan L, Mathews KS, Melamed ML (2020). Factors associated with death in critically ill patients with coronavirus disease 2019 in the US. JAMA Intern Med.

[6] Hirsch JS, Ng JH, Ross DW, Sharma P, Shah HH, Barnett RL (2020). Acute kidney injury in patients hospitalized with COVID-19. Kidney Int.

[7] Nogueira SÁR, Oliveira SCS, Carvalho AFM, Neves JMC, Silva LSVD, Silva Junior GBD Renal changes and acute kidney injury in COVID-19: a systematic review. Rev Assoc Med Bras (1992). 2020;66Suppl.

[8] Khwaja A (2012). KDIGO clinical practice guidelines for acute kidney injury. Nephron Clin Pract.

[9] Charlson ME, Pompei P, Ales KL, MacKenzie CR (1987). A new method of classifying prognostic comorbidity in longitudinal studies: development and validation. J Chronic Dis.

[10] Seymour CW, Liu VX, Iwashyna TJ (2016). Assessment of clinical criteria for sepsis: for the third international consensus definitions for sepsis and septic shock (Sepsis-3). JAMA.

[11] Grasselli G, Greco M, Zanella A, Albano G, Antonelli M, Bellani G (2020). Risk factors associated with mortality among patients with COVID -19 in intensive care units in Lombardy, Italy. JAMA Intern Med.

[12] Xie J, Wu W, Li S, Hu Y, Hu M, Li J (2020). Clinical characteristics and outcomes of critically ill patients with novel coronavirus infectious disease (COVID-19) in China: a retrospective multicenter study. Intensive Care Med.

[13] Gabarre P, Dumas G, Dupont T, Darmon M, Azoulay E, Zafrani L (2020). Acute kidney injury in critically ill patients with COVID-19. Intensive Care Med.

[14] Ng JH, Bijol V, Sparks MA, Sise ME, Izzedine H, Jhaveri KD (2020). Pathophysiology and pathology of acute kidney injury in patients with COVID-19. Adv Chronic Kidney Dis.

[15] Cheng Y, Luo R, Wang K, Zhang M, Wang Z, Dong L (2020). Kidney disease is associated with in-hospital death of patients with COVID-19. Kidney Int.

[16] Beier K, Eppanapally S, Bazick HS, Chang D, Mahadevappa K, Gibbons FK (2011). Elevation of blood urea nitrogen is predictive of long-term mortality in critically ill patients independent of “normal” creatinine. Crit Care Med.

[17] Wang L, Li X, Chen H, Yan S, Li D, Li Y (2020). Coronavirus Disease 19 infection does not result in acute kidney injury: an analysis of 116 hospitalized patients from Wuhan, China. Am J Nephrol.

[18] Massari F, Scicchitano P, Ciccone MM, Caldarola P, Aspromonte N, Iacoviello M (2019). Bioimpedance vector analysis predicts hospital length of stay in acute heart failure. Nutrition.

[19] Bowe B, Cai M, Xie Y, Gibson AK, Maddukuri G, Al-Aly Z (2020). Acute kidney injury in a national cohort of hospitalized us veterans with COVID-19. Clin J Am Soc Nephrol.

[20] Kolhe NV, Fluck RJ, Selby NM, Taal MW (2020). Acute kidney injury associated with COVID-19: A retrospective cohort study. PLoS Med.

[21] Rees EM, Nightingale ES, Jafari Y, Waterlow NR, Clifford S, Pearson CA B (2020). COVID-19 length of hospital stay: a systematic review and data synthesis. BMC Med.

[22] Eriksson KE, Campoccia-Jalde F, Rysz S, Rimes-Stigare C (2021). Continuous renal replacement therapy in intensive care patients with COVID-19; survival and renal recovery. J Crit Care.

[23] Gupta S, Coca SG, Chan L, Melamed ML, Brenner SK, Hayek SS (2021). AKI treated with renal replacement therapy in critically ill patients with COVID-19. J Am Soc Nephrol.

[24] Lu JY, Hou W, Duong TQ (2021). Longitudinal prediction of hospital-acquired acute kidney injury in COVID-19: a two-center study. Infection.

[25] Procaccini FL, Alcázar Arroyo R, Albalate Ramón M, Torres Aguilera E, Martín Navarro J, Ryan Murua P (2021). Acute kidney injury in 3182 patients admitted with COVID-19: a single-center, retrospective, case-control study. Clin Kidney J.

[26] Serafim RB, Póvoa P, Souza-Dantas V, Kalil AC, Salluh JIF (2021). Clinical course and outcomes of critically ill patients with COVID-19 infection: a systematic review. Clin Microbiol Infect.

[27] De Carvalho H, Letellier T, Karakachoff M, Desvaux G, Caillon H, Papuchon E (2021). Hyponatremia is associated with poor outcome in COVID-19. J Nephrol.

[28] Berni A, Malandrino D, Corona G, Maggi M, Parenti G, Fibbi B (2021). Serum sodium alterations in SARS CoV-2 (COVID-19) infection: impact on patient outcome. Eur J Endocrinol.

[29] Deodatus JA, Kooistra SA, Kurstjens S, Mossink JCL, van Dijk JD, Groeneveld PHP (2021). Lower plasma calcium associated with COVID-19, but not with disease severity: a two-centre retrospective cohort study. Infect Dis (Lond).

[30] Yan Q, Zuo P, Cheng L, Li Y, Song K, Chen Y (2021). Acute kidney injury is associated with in-hospital mortality in older patients with COVID-19. J Gerontol A Biol Sci Med Sci.

[31] Fisher M, Neugarten J, Bellin E, Yunes M, Stahl L, Johns TS (2020). AKI in hospitalized patients with and without COVID-19: a comparison study. J Am Soc Nephrol.

[32] Lertdumrongluk P, Streja E, Rhee CM, Moradi H, Chang Y, Reddy U (2019). Survival advantage of African American dialysis patients with end-stage renal disease causes related to APOL1. Cardiorenal Med.

[33] Tuty Kuswardhani RA, Henrina J, Pranata R, Anthonius Lim M, Lawrensia S, Suastika K (2020). Charlson comorbidity index and a composite of poor outcomes in COVID-19 patients: a systematic review and meta-analysis. Diabetes Metab Syndr.

[34] See YP, Young BE, Ang LW, Ooi XY, Chan CP, Looi WL (2021). Risk factors for development of acute kidney injury in covid-19 patients: a retrospective observational cohort study. Nephron.

[35] Palaiodimos L, Kokkinidis DG, Li W, Karamanis D, Ognibene J, Arora S (2020). Severe obesity, increasing age and male sex are independently associated with worse in-hospital outcomes, and higher in-hospital mortality, in a cohort of patients with COVID-19 in the Bronx, New York. Metabolism.

[36] Richardson S, Hirsch JS, Narasimhan M, Crawford JM, McGinn T, Davidson KW (2020). Presenting characteristics, comorbidities, and outcomes among 5700 patients hospitalized with COVID-19 in the New York City area. JAMA.

[37] Chand S, Kapoor S, Orsi D, Fazzari MJ, Tanner TG, Umeh GC (2020). COVID-19-associated critical illness-report of the first 300 patients admitted to intensive care units at a New York City medical center. J Intensive Care Med.

[38] Taher A, Alalwan AA, Naser N, Alsegai O, Alaradi A (2020). Acute kidney injury in COVID-19 pneumonia: a single-center experience in Bahrain. Cureus.

[39] Lu JY, Babatsikos I, Fisher MC, Hou W, Duong TQ (2021). Longitudinal clinical profiles of hospital vs. community-acquired acute kidney injury in COVID-19. Front Med (Lausanne).

